# Photo Quiz: A fatal infectious case subsequent to pecking by a rooster

**DOI:** 10.1128/jcm.01300-25

**Published:** 2026-03-11

**Authors:** Yuansu Jiang, Siru Zhou, Daohong Zhou, Weiping Lu

**Affiliations:** 1 Department of Clinical Laboratory, Daping Hospital, Army Medical University 66307, Chongqing, People's Republic of China; 2 War Trauma Medical Center, State Key Laboratory of Trauma and Chemical Poisoning, Daping Hospital, Army Medical University 66307, Chongqing, People's Republic of China; Mayo Clinic Minnesota, Rochester, Minnesota, USA

**Keywords:** etiological research, *Clostridium perfringens*, *Clostridium septicum*, gas gangrene, rooster pecking

## PHOTO QUIZ 

**Fig 1:**
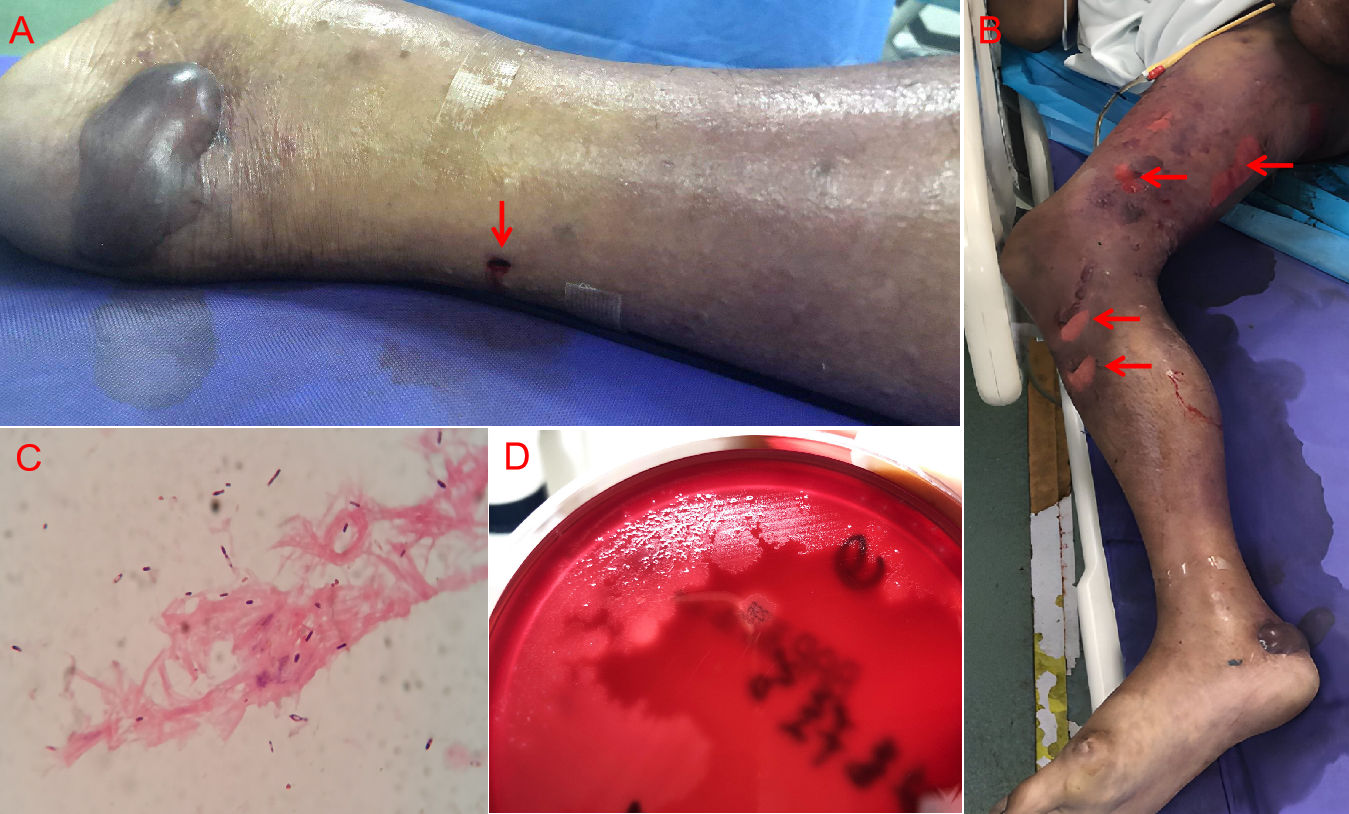
(**A**) The pecking wound on the right lower limb (at the red arrow). (**B**) Extensive severe damage of the right lower limb 2 days after the injury, the arrows point to ruptured bullae. (**C**) Multiple gram-positive bacilli in deep tissue effusion with Gram staining (×1,000). (**D**) Colony morphology on the Colombian blood plate (Anaerobic culture for 48 h).

An 85-year-old male farmer presented with swelling, pain, and mobility impairment in the right limb after being pecked by a rooster 2 days before admission to our hospital (Fig. 1A). Panel A shows the pecking wound on the right lower limb at the red arrow). Following the peck injury, the minor wound without obvious redness or swelling was treated with disinfection and cephalexin at a local hospital but showed no improvement. He was transferred to our emergency department immediately due to the rapidly spreading pain and scattered small blisters on the right lower limb. Physical examination revealed extensive crepitation of the body and right lower limb; scattered exfoliative dermatitis with malodorous serosanguineous exudate; swelling of the perineum, scrotum, and the right lower limb with cyanosis; disappearance of pulsation of the right dorsalis pedis; and ochrodermia with scattered blood blisters (Fig. 1B). Imaging examination showed gas accumulation in multiple soft tissues, with loss of normal muscle tissue morphology. The patient received surgical debridement immediately. Extensive myonecrosis of the right lower limb, perineum, buttocks, and right chest wall was found. Blister puncture fluid, necrotic tissue, and deep tissue effusion taken during debridement were further examined for pathogenic microorganisms. Gram-positive bacilli with spore formation were revealed by microscopic examination (Fig. 1C). After 48 h of anaerobic incubation, the swarming, gray-white colonies with beta-hemolytic rings appeared on Columbia sheep blood agar (Fig. 1D). After surgery, the patient was admitted to the intensive care unit and received clindamycin, combined with meropenem and continuous renal replacement therapy. Unfortunately, the patient opted for self-discharge due to personal circumstances and died the following day.

What is your diagnosis?

## ANSWER TO PHOTO QUIZ

The patient was diagnosed with gas gangrene. Growth of colonies from the surgical specimen was further identified as *Clostridium septicum (C. septicum*) by matrix-assisted laser desorption/ionization time-of-flight mass spectrometry (MALDI-TOF MS) using the VITEK MS system (bioMérieux, France). The testing was performed according to the manufacturer’s protocol, which involves applying the extracted samples onto the target slide, followed by overlaying them with the matrix solution (α-cyano-4-hydroxy-cinnamic acid) and subsequent analysis by the instrument. The identification of *C. septicum* was achieved with a high confidence score of 99.9%.

Gas gangrene, or clostridial myonecrosis, is an infection of muscle tissue necrosis, interstitial hemorrhage, and edema caused by strictly anaerobic Clostridium species, which mainly include *Clostridium perfringens* and *C. septicum* (1). Gas gangrene progresses rapidly, and the margins of necrotic tissue often expand at a rate of several centimeters per hour without prompt and appropriate antibiotic treatment (2). *C. septicum* is a motile, gas-toxin-producing, spore-forming, anaerobic gram-positive bacillus, leading to rapidly progressive gas gangrene by alpha-toxin (3). It has been reported to be present in the environment, soil, and normal appendices (4). Most *C. septicum* infections are spontaneous, and a few are caused by trauma (5). It is noteworthy that spontaneous *C. septicum* infections are frequently associated with gastrointestinal malignancies and hematological disorders, warranting further diagnostic evaluation.

Diagnosis of *C. septicum* infection lies on clinical signs and imaging, with confirmation by histopathology and etiological testing. The disease is characterized by acute, rapidly progressive tissue necrosis. Imaging reveals soft-tissue gas and myonecrosis. Treatment mandates immediate surgical debridement, antibiotics, and supportive care. Surgical intervention should not be delayed pending culture results. Concurrently, prompt initiation of penicillin G combined with clindamycin is essential for antimicrobial coverage and toxin suppression. The mortality rate of Clostridial myonecrosis approaches 100% if left untreated (1); rapid and accurate identification is the key to improving the prognosis.

We reported a rare fatal case of severe invasive infection caused by *C. septicum* from the environment through a pecking wound by a rooster. The rooster transmitted *C. septicum* from soil via a narrow, penetrating peck wound. Inadequate wound management resulted in anaerobic infection. Crucially, trauma in the polluted environment requires a high level of vigilance against infection by special pathogens. Etiological research and rapid identification and detection are of great significance for early diagnosis and precise treatment of pathogenic microorganism infection.
